# Rotaphone-CY: The Newest Rotaphone Model Design and Preliminary Results from Performance Tests with Active Seismic Sources

**DOI:** 10.3390/s21020562

**Published:** 2021-01-14

**Authors:** Johana Brokešová, Jiří Málek, Jiří Vackář, Felix Bernauer, Joachim Wassermann, Heiner Igel

**Affiliations:** 1 Department of Geophysics, Faculty of Mathematics and Physics, Charles University, V Holešovičkách 2, 180 00 Prague, Czech Republic; 2 Department of Seismotectonics, Institute of Rock Structure and Mechanics, Czech Academy of Sciences, V Holešovičkách 41, 182 09 Prague, Czech Republic; malek@irsm.cas.cz (J.M.); vackar@irsm.cas.cz (J.V.); 3 Department of Earth and Environmental Sciences, Ludwig Maximilian University of Munich, Theresienstr. 41, D-80333 Munich, Germany; fbernauer@geophysik.uni-muenchen.de (F.B.); jowa@geophysik.uni-muenchen.de (J.W.); igel@geophysik.uni-muenchen.de (H.I.)

**Keywords:** seismic rotation, rotational seismometer, Rotaphone, seismic array, rotation-to-translation relations, field experiment

## Abstract

Rotaphone-CY is a six-component short-period seismograph that is capable of the co-located recording of three translational (ground velocity) components along three orthogonal axes and three rotational (rotation rate) components around the three axes in one device. It is a mechanical sensor system utilizing records from elemental sensors (geophones) arranged in parallel pairs to derive differential motions in the pairs. The pairs are attached to a rigid frame that is anchored to the ground. The model design, the latest one among various Rotaphone designs based on the same principle and presented elsewhere, is briefly introduced. The upgrades of the new model are a 32-bit A/D converter, a more precise placing of the geophones to parallel pairs and a better housing, which protects the instrument from external electromagnetic noise. The instrument is still in a developmental stage. It was tested in a field experiment that took place at the Geophysical Observatory in Fürstenfeldbruck (Germany) in November 2019. Four Rotaphones-CY underwent the huddle-testing phase of the experiment as well as the field-deployment phase, in which the instruments were installed in a small-aperture seismic array of a triangular shape. The preliminary results from this active-source experiment are shown. Rotaphone-CY data are verified, in part, by various approaches: mutual comparison of records from four independent Rotaphone-CY instruments, waveform matching according to rotation-to-translation relations, and comparison to array-derived rotations when applicable. The preliminary results are very promising and they suggest the good functionality of the Rotaphone-CY design. It has been proved that the present Rotaphone-CY model is a reliable instrument for measuring short-period seismic rotations of the amplitudes as small as 10${}^{-7}$ rad/s.

## 1. Introduction

Rotational seismology and seismometry are relatively new seismological disciplines dealing with rotational ground motions. Since their establishment in the 2000s, they have been attracting increased attention in the seismological community [1,2]. For a broader state-of-the-art review, please refer to the companion paper by Brokešová and Málek, this issue [3].

In this paper, by seismic rotation, we mean rotation rate Ω directly related to the curl of ground velocity v. In the right-handed Cartesian coordinate system x,y,z (*x*-axis positive to the East, *y*-axis positive to the North, *z*-axis positive upwards, the origin on the Earth’s surface), ground velocity v can be decomposed into three translational components $v_{x}$, $v_{y}$, and $v_{z}$. Seismic rotation Ω is then decomposed into rotational components $\Omega_{x}$, $\Omega_{y}$, and $\Omega_{z}$, which represent rotation rates around the corresponding coordinate axes. At the Earth’s surface, the expressions for them simplify thanks to the free-surface boundary conditions and they read

(1) $$\begin{array}{ccc} \Omega_{x} & = & \frac{\partial v_{z}}{\partial y}\\ \Omega_{y} & = & -\frac{\partial v_{z}}{\partial x}\\ \Omega_{z} & = & \frac{1}{2}\left(\frac{\partial v_{x}}{\partial y}-\frac{\partial v_{y}}{\partial x}\right). \end{array}$$

According to the sign convention we have adopted, rotation rates are positive counter-clockwise in accordance with the right-handed ’rule of thumb’, as suggested by Evans [4].

Distant-source seismic wavefields can usually be approximated by plane waves that propogate along the Earth’s surface with an apparent velocity *c*. Under the assumption of a single plane wave, seismic rotational and translational components are related to each other via well known rotation-to-translation relations (R-TRs) [5,6,7], the simplest in the rotated Cartesian coordinates ξ,η,z, with ξ being the radial axis (along the surface, positive in the wave propagation direction) and η being the transverse axis (along the surface, parallel to the wavefront and complementing the coordinates to a right-handed system). The distant-source R-TRs relate the rotation rate components to the components of ground acceleration a (time derivative of v) and read

(2) $$\begin{array}{ccc} \Omega_{\xi } & = & 0\\ \Omega_{\eta } & = & \frac{1}{c}a_{z}\\ \Omega_{z} & = & -\frac{1}{2c}a_{\eta }. \end{array}$$

These R-TRs have been widely applied in many studies in order to determine propagation direction (true back azimuth) [8], apparent phase velocity *c* [7], or even to estimate Love-wave velocity dispersion [9], with all of that from a single station measurement. Those studies utilized records of rotational rates from teleseismic earthquakes that were measured by the so-called ring-laser gyroscopes [10,11] based on the Sagnac effect [12]. Ring lasers are very sensitive rotational sensors that dominated rotational seismometry in the 2000s, and new and more capable instruments of this type are still being constructed—an example is the large four-component ROMY ring laser that was installed in 2017 at the Geophysical Observatory in Fürstenfeldbruck (GOF) near München (Germany) under the auspices of Ludwig-Maximilians-Universität München [13,14,15]. However, a disadvantage of such highly sensitive rotational sensors is their very costly installation, operation, and maintenance. They are not designed for field deployment. Portable, field-deployable rotational sensors that are based on different physical principles started to appear, especially in the last decade, being developed independently by various scientific teams or companies [16,17,18,19]. The authors of this paper have been developing the so-called Rotaphones [3,20,21,22,23,24], also falling into this category, since 2008. However, there is a very important feature discriminating Rotaphone from the other field-deployable seismic rotational sensors: Rotaphone, despite its name, is a six-component (6C) instrument that was designed for recording both rotational and translational components by one and the same device. Moreover, in the 6C Rotaphone records, rotation rates are free of translational velocities and vice versa. Among various model designs, Rotaphone-CY is the latest one and its performance is the focus of the present study.

There is no principal reason not to employ Equation (2) for rotational data from portable, field-deployable sensors, whenever the plane wave assumption is justified. Moreover, they offer another application, not much exploited up to now: verifying the recorded rotational waveforms against the acceleration waveforms (that are unquestionable, as they are recorded by classical seismographs that are used in traditional seismology).

Most, if not all, portable, field-deployable, rotational sensors are clearly much less sensitive than the ring-laser gyroscopes, which predestinates them to be deployed at local/regional distances (i.e., to record ground motions from relatively proximal sources). However, for a proximal source, the plane wave assumption may not be acceptable. Brokešová and Málek [25] derived generalized R-TRs under the assumption of a spherical wave generated by a directional point source. In addition to the ground acceleration terms, those relations also contain ground velocity terms, which, in general, cannot be neglected at a small epicentral distance and/or at higher frequencies and/or in regions of rapid changes of amplitudes. The generalized R-TRs read

(3) $$\begin{array}{ccc} \Omega_{\xi } & = & C_{11}v_{z},\\ \Omega_{\eta } & = & C_{21}v_{z}+C_{22}a_{z},\\ \Omega_{z} & = & C_{31}^{\eta }v_{\eta }+C_{31}^{\xi }v_{\xi }+C_{32}a_{\eta }. \end{array}$$

The coefficients are specified by Brokešová and Málek [25]. For this study, it is sufficient to note that $C_{22}=1/c$, $C_{32}=-1/\left(2c\right)$ and the remaining coefficients are related to spatial derivatives of amplitude and/or (in the case of $C_{21}$ and $C_{31}$) to the wavefront curvature. Based on the authors’ experience, the presence of velocity terms in R-TRs is often manifested by a time shift between the left-hand and right-hand sides of the equations. Thus, R-TRs still can help to verify the rotational records, although they are only approximate in inhomogeneous media.

The onset of various portable rotational sensors has given rise to field and laboratory experiments that aimed at comparing their records with those of ring-lasers or among themselves [3,18,26,27]. In November 2019, an extensive comparative rotation sensor test experiment was organized at GOF. The experiment is described by Bernauer et al. [28], in this issue. More than twenty field-deployable rotational sensors were involved, including four Rotaphone-CY instruments that were, however, in a development stage at that time. A part of the development phase is a precise calibration of Rotaphone as a system of elemental sensors (geophones), without which the Rotaphone records are not correct. The calibration phase was originally scheduled to have taken place in the specialized USGS Albuquerque Seismological Laboratory (ASL), U.S.A., equipped with excellent facilities for the purpose, in April 2020. The envisaged calibration of the four Rotaphone-CY instruments at ASL could not be conducted due to the severe restrictions adopted worldwide as a response to the COVID-19 pandemic in 2020. As a substitute solution of the situation, the facilities that are available at the Institute of Rock Structure and Mechanics, Czech Academy of Sciences (Prague, Czech Republic) have been used instead of the ASL specialized equipment. Those facilities are able to calibrate Rotaphone-CY only up to ∼20 Hz, far below the upper frequency limit of the instrument. Thus, at the time of writing this paper, the Rotaphone records must be considered as preliminary, by reason of the frequency range limitation, which is incapable of a full-fledged comparison with records from the other rotational sensors involved in the GOF experiment. Only a limited-extent comparison can be shown in this journal issue [28]. The final results will be published elsewhere once the calibration is completed.

The present study focuses on a comparison of records from the GOF experiment among the four Rotaphones-CY as well as on their verification either with the help of R-TRs or the method of array-derived rotations (ADR) [29,30]. The companion paper describes the ADR method in detail, including its severe applicability limitations [3].

## 2. Rotaphone-CY

Rotaphones are mechanical sensor systems that are capable of measuring six components of seismic ground motion: three orthogonal translational components (ground velocities) and three rotation rates around the same three axes. Rotaphones have been developed since 2008 and they exist in various model designs, of which Rotaphone-CY is the latest one. The basic idea underlying the design of the instruments is that they measure spatial ground velocity gradients by means of differential motions that were recorded by parallel pairs of elemental sensors (geophones) that are separated by a distance that is two or three orders of magnitude smaller than the wavelength of the measured wavefield. The separation distance is typically few tens of cm and so only high-sensitivity geophones, thoroughly calibrated, allow for such differential sensing. The geophones are mounted to a rigid (metal) frame that is anchored to the ground. The whole instrument is supposed to move as a rigid body when seismic waves are passing through the site. For a detailed description of the Rotaphone principle, please refer to the companion paper [3] or older papers [20,21,23,25].

Even though the elemental geophones that are used in the system (in one Rotaphone instrument) are of the same type, made by the same manufacturer or even belonging to the same batch, their instrument characteristics (response functions) are never exactly identical and equal to the manufacturer-specified response of the given geophone type. Even small variations in response functions may significantly influence the differences in records over such a small separation distance. Thus, an accurate and careful calibration of the individual geophones and the whole instrument is unavoidable. It consists of two parts. First, in-lab pre-calibration that can be done only once for the given sensor and that makes it possible to compensate for the most significant differences in individual responses. This pre-calibration was not completed at the time of writing this paper for an objective reason of the strict ban on travelling related to COVID-19 and only a preliminary pre-calibration up to ∼20 Hz was used instead. Second, the so-called in-situ calibration was performed simultaneously with data processing, thus being an integral part of each measurement. The in-situ calibration [21,25] relies on the redundancy of rotational records thanks to the arrangement of geophone pairs on the frame (more than one pair for each rotational component) and rigidity of the frame. This type of calibration is not less important than the pre-calibration, as it can compensate for fine differences due to varying physical conditions at the site (temperature, air pressure, humidity, magnetic field variations, etc.) and to geophone aging.

**Figure 1:**
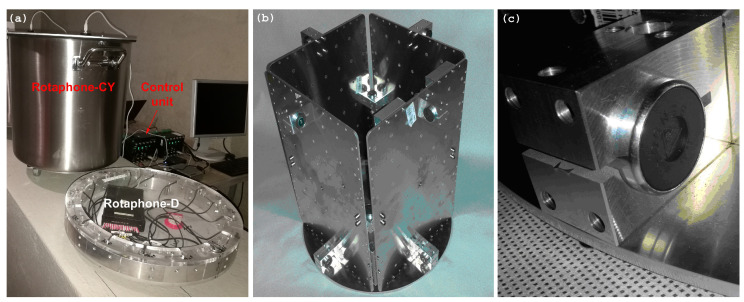
Rotaphone-CY: an overall view of the instrument (compared to the ancestor six-component (6C) Rotaphone-D design) and its control unit (**a**), inner frame with geophones (**b**), and a detail of a horizontal geophone SM-6 mounted to the inner frame (**c**).

Figure 1 shows Rotaphone-CY, together with the preceding model design, 6C Rotaphone-D. It contains two pairs of vertical geophones and four pairs of horizontal geophones (SM-6 by ION Sensor Nederland b.v.) that are attached to the inner frame (Figure 1b,c), arranged in an analogous way as in the older model, Rotaphone-C [22], as shown in the companion paper [3] (Figure 4 in that paper). Its normalized transfer function, both translational ground velocity and rotation rate, is also the same (Figure 5 in the companion paper [3]). When comparing the latest model Rotaphone-CY to the older design, Rotaphone-C, the main differences are (1) geophones SM-6 with higher sensitivity, (2) a 16-channel 32-bit A/D transducer by Embedded Electronics & Solutions, Ltd., and (3) a compact and waterproof cylindrical housing. Table 1 presents the parameters of the instrument that is derived from the manufacturer specifications. The upper frequency limit is given by the frame’s natural frequency (first resonance mode frequency), because only up to this frequency the frame moves as a rigid body. As mentioned above, at the time of writing this paper, the upper limit had to be reduced to ∼20 Hz because of the incomplete in-lab pre-calibration. The lower limit represents the manufacturer-specified lower frequency limit of the geophones. It will be examined while utilizing a specialized equipment of ASL as soon as the external circumstances allow.

The resolutions shown in Table 1 are derived from the geophone sensitivity and the parameters of the A/D transducer. The values are only theoretical and noise-free. In reality, noise is always present, both natural and instrument-related.

## 3. Huddle Test

**Figure 2:**
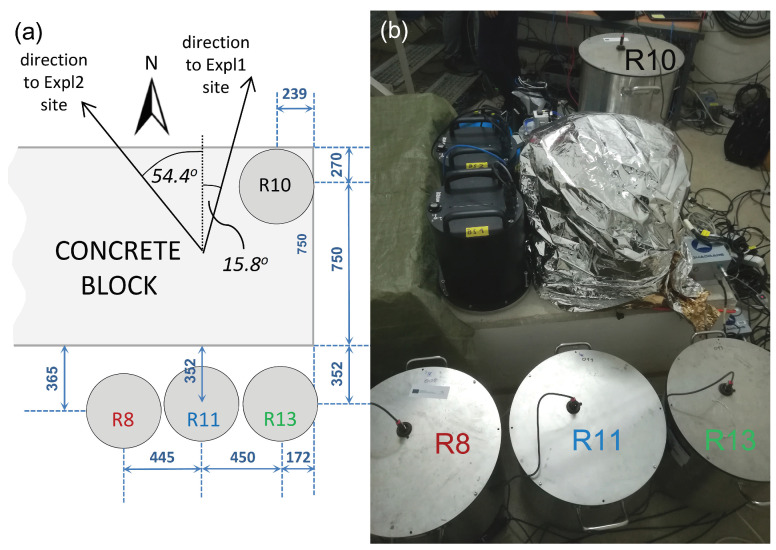
Four Rotaphone-CY instruments (R8, R10, R11, and R13) in the huddle test: scheme showing the positions (**a**) and photograph (**b**). The color coding used to mark individual Rotaphone-CY instruments is kept throughout the manuscript in order to distinguish their records. The numbers refer to distances in mm to the centers of the instruments.

In the first part of the GOF experiment, all of the sensors were placed into a seismic vault (the vault in which the German Regional Seismic Network station FUR is situated) for huddle testing. The vault is approximately 5 m deep and, in its base, there is a concrete block, seismically decoupled from the rest of the building. During the huddle test, 26 instruments that were involved in the GOF experiment were installed in the vault for two days, as close to each other as possible. In this paper, due to the frequency range limitation, we do not compare the Rotaphone results with those from the other instruments. We only present Rotaphone data and focus on their mutual comparison. One of the Rotaphone-CY instruments was seated on the top of the concrete block, while the remaining three were placed directly on the ground, in a very short distance from the block (Figure 2).

The purpose of the huddle test was two-fold: (1) a continuous measurement overnight when cultural noise is at a minimum in order to estimate the lowest ambient and/or instrument-related noise level and (2) to compare the records of the multiple instruments located as close as possible to each other from an active source in order to view the recorded data for possible anomalies and check the functionality of the instruments and their consistency.

**Figure 3:**
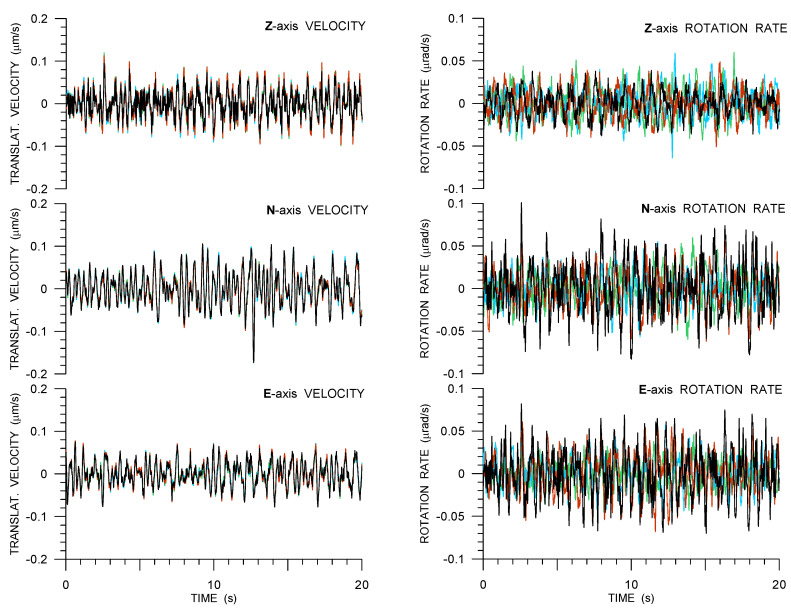
Twenty seconds of overnight noise as recorded by four 6C Rotaphone-CY instruments (R8—red, R10—black, R11—light blue, and R13—green) in the huddle test.

All of the Rotaphone data presented in this section are low-pass filtered while using the causal eight-pole Butterworth filter with the cut-off frequency of 20 Hz. Figure 3 illustrates the noise measurement. The figure shows an example of a 20-s time interval of the overnight noise in all six components that were recorded in the vault on 19 November 2019. It allows for us to understand the nature of the noise and estimate from below the least detectable real ground motions at the site.

Despite the resolution values recorded in Table 1, in the GOF experiment we could not record reasonable ground motions with amplitudes lower than 10${}^{-7}$ in both types of motion. Translational noise records (part (a) of Figure 3) are almost perfectly correlated for all four instruments, which suggests that the noise is natural (seismic) and not instrument-related (self-noise). The rotational noise from all of the instruments is much less correlated (Table 2). It is difficult to say to what extent it reflects the self-noise of Rotahone-CY as a rotational sensor, because rotation rates as well as a natural rotational noise may change rapidly in space. Table 2 illustrates that the rotational noise that is recorded by the four instruments is not totally random and it may be, at least in part, also of a natural origin.

### Active-Source Results

In the active part of the huddle test, two small-size explosions were fired in the vicinity of the vault, as shown in Table A1.

**Figure 4:**
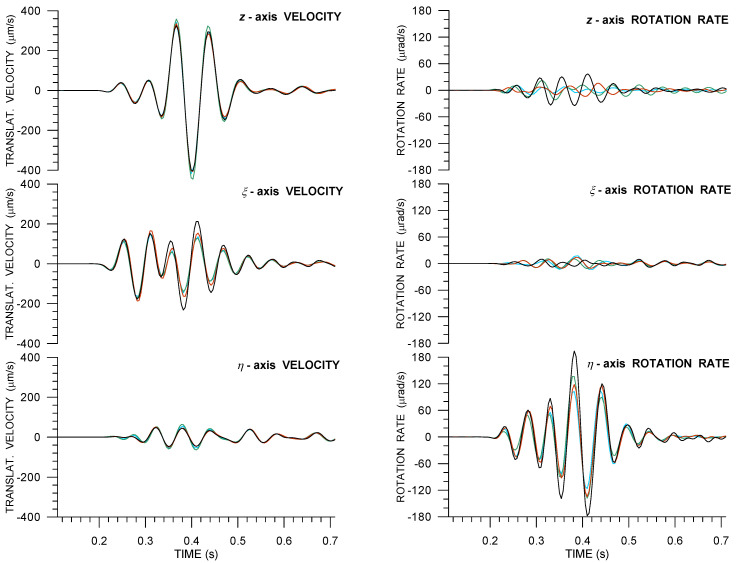
A small-size explosion (Expl2 in Table A1) at a distance of 52 m from the vault as recorded by four 6C Rotaphone-CY instruments (R8—red, R10—black, R11—light blue, and R13—green) in the huddle test. Horizontal components are rotated into radial and transverse directions according to the geometrical azimuth of 305.6${}^{\circ }$.

As an example of Rotaphone records in this part of the experiment, Figure 4 shows a comparison of 6C records that was made by all four Rotaphone-CY instruments from the blast utilizing 500 g of explosives at a distance of 52 m from the vault in an azimuth of 305.6${}^{\circ }$ from N (the explosion is designated as Expl2 in Table A1). Note that the same event is presented in the paper by Bernauer et al. in this issue [28] where Rotaphone records are shown together with the records from the other rotational sensors that are involved in the GOF experiment. The substantial difference between Figure 4 and relevant figures in that paper is that, here, we show all six components (i.e., rotational records are supplemented by translational ones). Moreover, instead of North and East components (that are useful when comparing just basic instrument performance characteristics), we display radial and transverse components, which we consider to be more meaningful from the point of view of wave propagation physics. For instance, Figure 4 clearly shows that the ξ-axis rotation rate is the least among all of the rotation rate components, which is in agreement with Equation (2) predicting $\Omega_{\xi }=0$. The explosion-induced ground velocity reached the peak value of 4 $\times 10^{-4}$ m/s along *z*-axis and peak rotation rate of 1.8 $\times \;10^{-4}$ rad/s around η-axis (i.e., tilting in radial direction). The signal-to-noise ratio (SNR) is very good in these records; it is approximately 210–1740 in translations and even 220–2080 in rotations (depending on the motion component). Records from R8, R11, and R13, the instruments standing side-by-side on the ground next to the concrete block, show significant similarity in all of the components, except the *z*-axis rotation rate (in which almost no fit is observed, obviously the result of significant mutual differences in horizontal translational components, Equation (1)). On the other hand, the differences among their records are not negligible, not even in translational components. We interpret the differences as a consequence of the vault building deformation. Records from R10, the instrument standing on the concrete block decoupled from the vault building, differ the most from the others, especially in the ξ-axis velocity and all of the rotational components.

**Figure 5:**
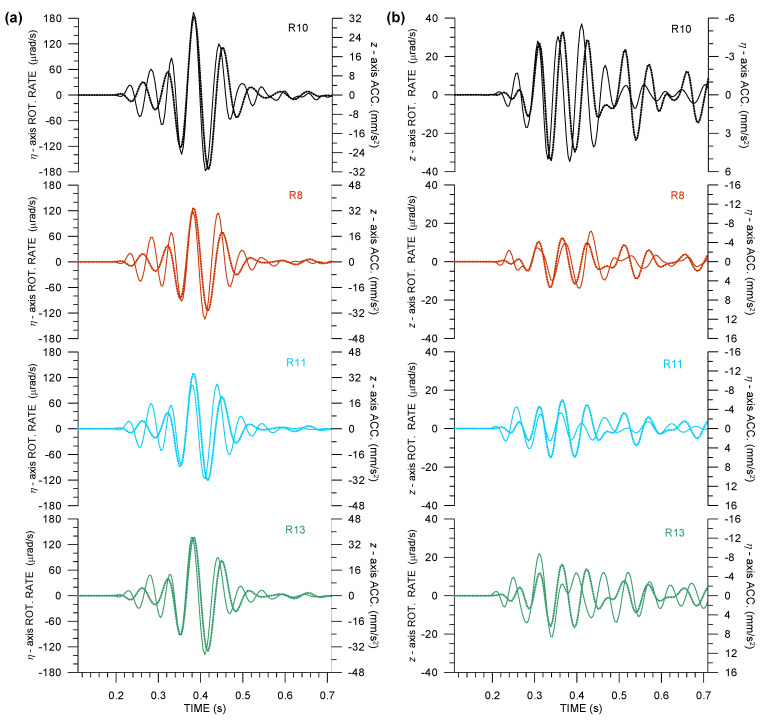
Waveform matching of the relevant rotation rate (solid) and ground acceleration (dotted) components in records of Expl2 in the huddle test from four 6C Rotaphone-CY instruments (R8—red, R10—black, R11—light blue, and R13—green): rotation rates around the transverse axis and vertical ground acceleration (**a**), and rotation rates around the vertical axis and transverse ground acceleration (**b**). The transverse axis is perpendicular to the geometrical azimuth of 305.6${}^{\circ }$.

As Equations (2) and (3) suggest, it is interesting to look at the waveform matching of the η-axis rotation rate and *z*-axis ground acceleration, as well as waveform matching of the *z*-axis rotation rate and η-axis ground acceleration, as in Figure 5.

Having the small source-receiver distance in mind, the waveform match is surprisingly good for the S-wave part of the seismograms shown in Figure 5a, and it is not so bad, even in Figure 5b, despite the striking differences in *z*-axis rotation rates, as recorded by the four Rotaphones (Figure 4b, top). Going into detail, the waveform match from R10 (installed on the concrete block decoupled from the vault building) differs significantly from the other instruments, both in amplitude ratios and the phase shift that is obvious in Figure 5b, top. Both of the features can be precisely related to the presence of the concrete block below R10 and in the closest vicinity of the remaining Rotaphones. Note that the prevailing frequencies in the Expl2 records reach ∼16 Hz. Taking a rough apparent velocity estimate derived from amplitude ratios in Figure 5 into account, we come to the conclusion that the prevailing apparent wavelength can be roughly 10–20 m, depending on the wave type. At such a scale, the concrete block represents a distinct inhomogeneity. Moreover, the vault, comparable in dimensions to the wavelength, undergoes deformation as seismic waves pass over, which certainly induces normal modes. Those oscillations are clearly manifested in the differences in the translational records, unexplainable otherwise for such a short distance between the instruments. Nevertheless, they influence seismic rotations to a much higher extent.

During the huddle test, another explosion, much weaker (150 g of explosives) and more distant (220 m from the vault), was fired at an azimuth of 15.8${}^{\circ }$ from N. It is designated as Expl1 in Table A1. Table 3 summarizes the quantities characterizing translational and rotational records from both explosions.

In the case of Expl1, the translational velocity amplitudes are approximately up to 18 times and rotational rate amplitudes, even up to 48 times weaker when compared to Expl2. Consequently, SNR is much worse—it goes down to 31 and 22 for translational and rotational components, respectively. Conclusions drawn from Expl1 are mostly similar to the case of Expl2, except that the degree of mutual similarity of rotational waveforms among the four instruments is much lower, definitely not due to the worse SNR. The presence of the rectangular block can still be the cause due to possible complex interference phenomena, different from the Expl2 case because of different propagation direction and a slightly different prevailing wavelength (resulting from a slightly different prevailing frequency). However, it was not possible to quantify the prevailing wavelength due to the poor matching of the relevant rotation rate and acceleration components according to Equations (2) or (3). Another interesting fact is that, in contrast to Expl2, the ξ-axis rotation rate is not the least of all the rotational components. Equation (3) suggests a possible cause in the influential presence of velocity terms in R-TRs. However, a more likely cause is the interference of various waves (including vault normal modes), overlapping in time. In such a case, Equations (2) and (3) cannot correspond to the real R-TRs, as they have been derived under the assumption of a single wave, either plane or spherical.

## 4. Triangular Small-Aperture Array

**Figure 6:**
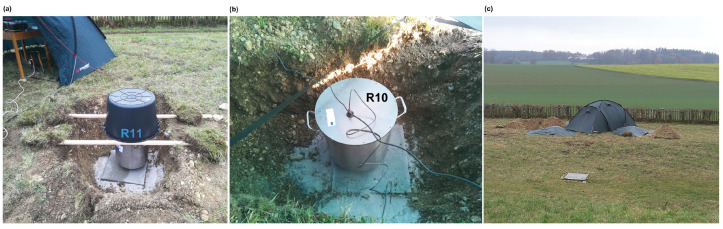
Field deployment of the Rotaphones-CY involved in the Geophysical Observatory in Fürstenfeldbruck (GOF) experiment: (**a**) Rotaphone R11, NW apex of the triangular array, before the waterproof foil was laid over, (**b**) the central Rotaphone R10, sheltered by a tent, and (**c**) the whole array as viewed from the North, R10 inside the tent, R8 and R11 on both sides, R13 hidden behind the tent.

**Figure 7:**
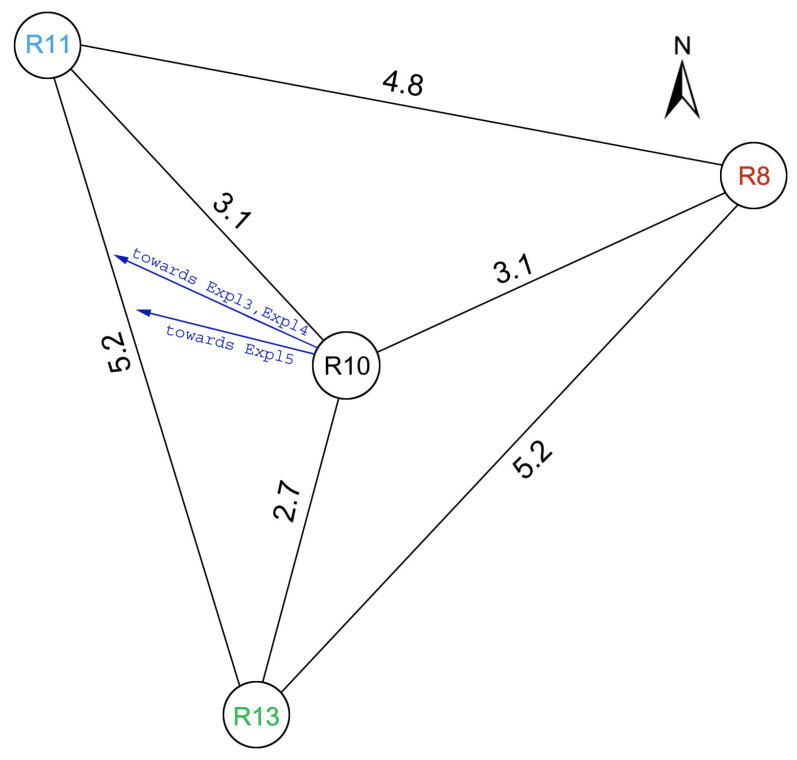
A detailed layout the Rotaphone-CY array. Numbers indicate the distance in meters between centers of the instruments.

After the huddle test, the instruments were moved outdoors to a space on the GOF premises and deployed there for several days. Four Rotaphones-CY were installed in shallow pits, half a meter deep, on a concrete slab (50 cm × 50 cm × 5 cm) at the bottom (Figure 6a,b). They were arranged to create a small triangular array with an aperture of ∼5 m with one instrument (R10) that is situated at the center of the triangle. The central instrument was protected by a tent housing all of the control units and hardware accessories of all the instruments (Figure 6b,c). The remaining three Rotaphones (R8, R11, and R13) were deployed at the apexes of the triangle at a distance of approximately 3 m from the center. They were equipped with plastic caps and waterproof plastic foils that extended well beyond the pit rims (Figure 6a,c). All the Rotaphones were supplied with GPS antennas used for time synchronization. Figure 7 shows a detailed configuration of the array.

### 4.1. Active-Source Results

During the active part of the GOF experiment, three small-size explosions (Table A1) at different distances from the central Rotaphone R10 were used as active sources. Namely, Expl3 at a distance of 452 m and azimuth of 294.0${}^{\circ }$, Expl4 at a distance of 676 m and azimuth of 293.0${}^{\circ }$, and Expl5 at a distance of 1020 m and azimuth of 284.8${}^{\circ }$. The amount of explosives was 1500 g in all three cases.

An interesting feature of the studied open-air measurements is the existence of a high-frequency sound wave propagating through the air at sound speed (∼340 m/s). It excites Rotaphone motion that is superposed on the measured ground motions, as seismic waves are relatively slow at the site (an uncosolidated alluvial basin with glacial deposits, [15]), which interferes with the measurements from the seismological point of view. Note that the sound wave was sufficiently decremented in the vault. In order to suppress this wave, a double cosine window between 1 and 15 Hz (taper width 10%) was applied to data in this section. Such a filter is not causal, which results in the presence of small oscillations prior to the first onset, but, on the other hand, the filter does not distort the waveforms significantly and maintains the time lags between the individual wave phases. However, for proximal sources, such phases often mix and overlap in time anyway.

During the data processing, it was discovered that one of the vertical geophones of R10 had suffered unspecified damage between the in-vault and field-deployment phases of the experiment. Therefore, it was necessary to remove the malfunctioning geophone from data processing (i.e., from in-situ calibration). The removal particularly affected the R10 records of transverse and radial rotation rate components, because the records were made using fewer geophone pairs than in the other Rotaphones. The records match well those from the other instrument in phase, but not in amplitude, as shown in the figures below. Therefore, $\Omega_{\xi }$ and $\Omega_{\eta }$ components from R10 were excluded from comparison with other Rotaphones as well as from apparent phase velocity and prevailing apparent wavelength estimates.

Table 4 summarizes data from all three above-mentioned explosions, analogous to Table 3 of the huddle test. As expected, the least peak values are observed for the most distant Expl5. The amplitudes are approximately up to 4× weaker when compared to Expl1 in Table 3. The SNR ratios do not drop below 20 for any component and any of the explosions so the seismograms are exemplary for the present study and allow for a more detailed analysis. A remarkable feature is that, when compared to the huddle test, in most of the field measurements the records from individual Rotaphones correlate much better between any two of the instruments, despite their mutual distance that is greater by one order of magnitude. This finding supports our hypothesis that the in-vault records were influenced a great deal by the vault normal modes, which may somewhat limit their use for the purpose of huddle testing.

Although the Pearson correlation coefficients presented in Table 4 show a relatively good similarity of the records from individual Rotaphones, in the case of the triangular array with an aperture of ∼5 m, they are not an optimum measure of similarity, because the records have clear mutual time shifts due to waves propagating at a speed of several hundred m/s in a given direction. After removing the shifts, the correlation coefficients are equivalent to the maxims of correlation function provided in Table 4 as $C_{T}^{\prime }$ and $C_{R}^{\prime }$. Such coefficients reveal a higher degree of similarity between Rotaphone records, especially rotational ones, which indicates a good functionality of the instruments.

**Figure 8:**
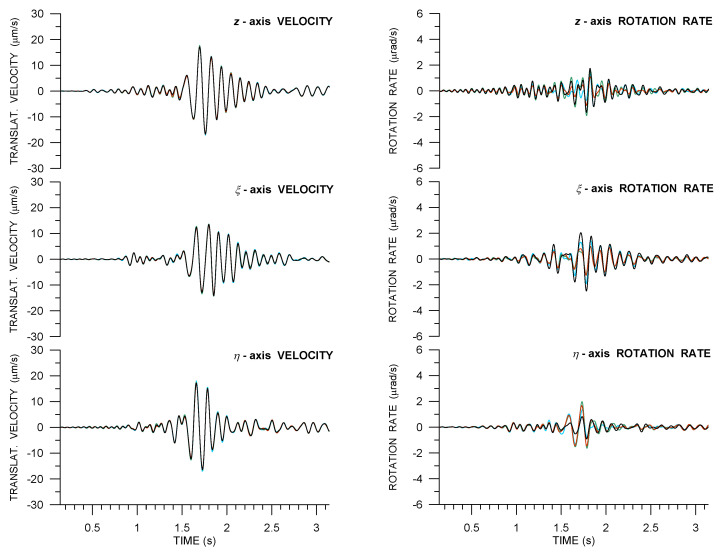
A small-size explosion (Expl3 in Table A1) at a distance of 452 m from R10 as recorded by four 6C Rotaphone-CY instruments (R8—red, R10—black, R11—light blue, and R13—green) in the active phase of the GOF experiment. The horizontal components are rotated into radial and transverse directions, according to the geometrical azimuth of 294.0${}^{\circ }$.

**Figure 9:**
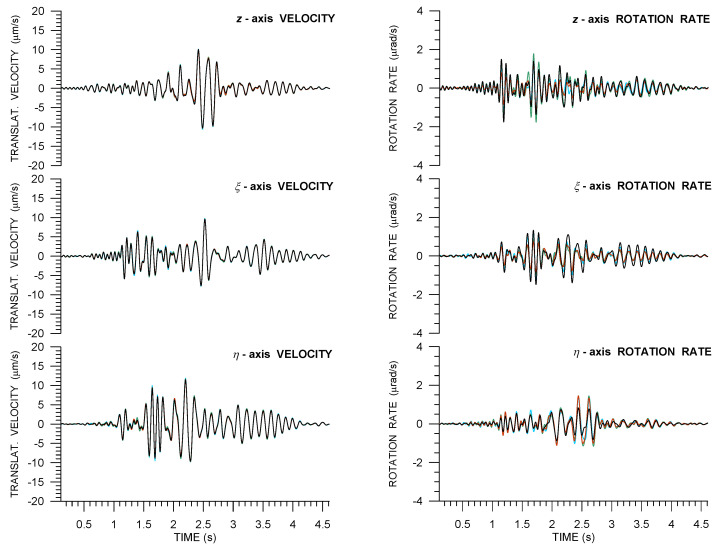
A small-size explosion (Expl4 in Table A1) at a distance of 676 m from R10 as recorded by four 6C Rotaphone-CY instruments (R8—red, R10—black, R11—light blue, and R13—green) in the active phase of the GOF experiment. Horizontal components are rotated into radial and transverse directions according to the geometrical azimuth of 293.0${}^{\circ }$.

**Figure 10:**
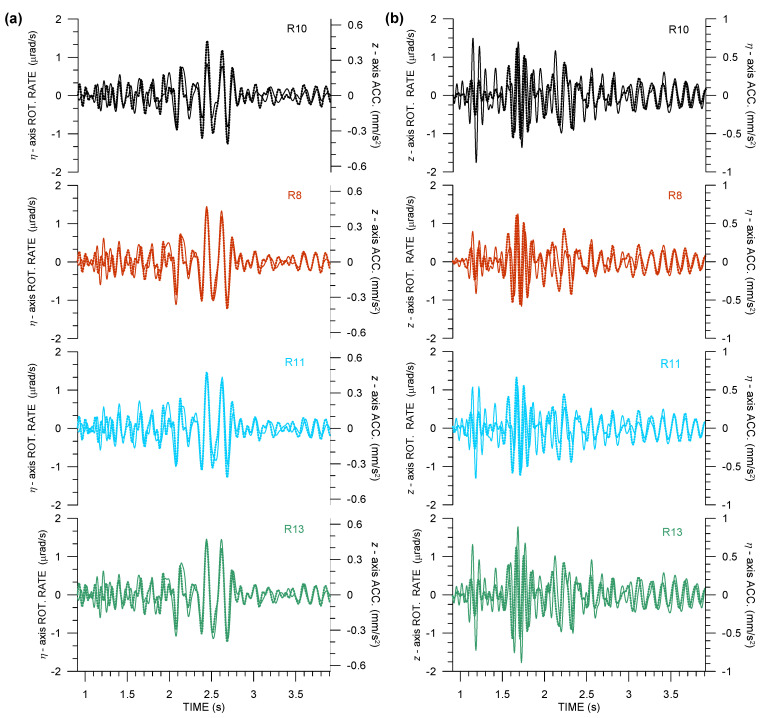
Waveform matching of the relevant rotation rate (solid) and ground acceleration (dotted) components in records of Expl4 in the active part of the GOF experiment from four 6C Rotaphone-CY instruments (R8—red, R10—black, R11—light blue, and R13—green): rotation rates around the transverse axis and vertical ground acceleration (**a**), and rotation rates around the vertical axis and transverse ground acceleration (**b**). The transverse axis is perpendicular to the geometrical azimuth of 293.0${}^{\circ }$.

**Figure 11:**
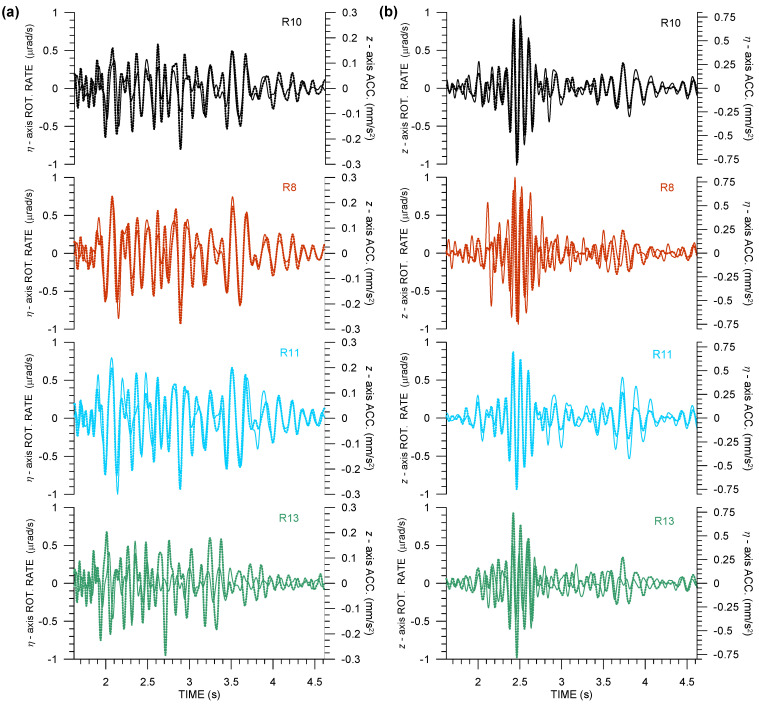
Waveform matching of the relevant rotation rate (solid) and ground acceleration (dotted) components in records of Expl5 in the active part of the GOF experiment from four 6C Rotaphone-CY instruments (R8—red, R10—black, R11—light blue, and R13—green): rotation rates that are around the transverse axis and vertical ground acceleration (**a**), and rotation rates around the vertical axis and transverse ground acceleration (**b**). The transverse axis is perpendicular to the geometrical azimuth of 285.0${}^{\circ }$.

Figure 8, Figure 9, Figure 10 and Figure 11 provide examples of detailed 6C records from the field-deployment part of the experiment, as well as examples of the waveform matching of relevant rotation rates and acceleration components according to Equations (2) and (3). The figures display records from all of the Rotaphones, i.e., including R10, in order to make clearer the nature of the technical issue discussed above.

Figure 8 and Figure 9 are fully analogous to Figure 4. They show the 6C records from explosions Expl3 and Expl4. Translational records from R10 (black curves) are fully consistent with records from the other three instruments. Additionally, the *z*-component rotation rate does not depart from the others, unlike the remaining two rotational components. In particular, whereas the R10 rotational records coincide in phase with the records from the other instruments, the $\Omega_{\xi }$ (radial) and $\Omega_{\eta }$ (transverse) amplitude from R10 is obviously not reliable, especially when taking the central position of R10 in the array into account. Therefore, those records are not considered in further analysis.

Figure 8 and Figure 9 both show a high degree of similarity in all rotational components (if we exclude the black curves from the comparison of $\Omega_{\xi }$ and $\Omega_{\eta }$ components), even higher for Expl4, as also confirmed by higher correlation in Table 4. A notable feature is that $\Omega_{\xi }$ is comparable in amplitude to $\Omega_{\eta }$ in all three cases, which rules out the plane-wave R-TRs that are described by Equation (2) and suggests that the real R-TRs are closer to those shown in Equation (3). The presence of velocity terms resulting from local inhomogeneities in the structure beneath the individual Rotaphones in the array may be the cause of a really poor correlation of $\Omega_{z}$ records for Expl5, see Table 4. The presence of velocity terms is manifested as slight phase shifts between the relevant rotation-rate and acceleration components, as clearly visible in the following two figures.

Figure 10 and Figure 11 are analogous to Figure 5 (that belong to the huddle-test Expl2). They show $\Omega_{\eta }-a_{z}$ and $\Omega_{z}-a_{\eta }$ waveform matching, as suggested by Equations (2) or (3). Note that the pair $\Omega_{\eta }-a_{z}$ is used in order to estimate the apparent phase velocity shown in Table 4. The estimated apparent phase velocity, as determined from different explosion measurements, varies from almost 600 m/s to approximately 300 m/s. The explanation of the differences is three-fold: (1) different seismogram phases, although all in the S-wave group, are associated with the peak value of $\Omega_{\eta }$ (or $a_{z}$) for different explosions, (2) the different phases may have different angles of incidence to the Earth’s surface—some of them probably propagate along the surface while others may incident in an oblique direction—and, (3) the peak phases may not be pure S waves; they can be mixed with waves from the P-wave group and surface-guided S waves to a various extent.

Figure 10a shows a good overall fit. Excluding R10, the best fit is achieved in the wave group between 2.3 s and 2.8 s containing the peak values of the whole seismograms. The peak values are used in the phase velocity estimate shown in Table 4. A comparably good fit with a similar amplitude ratio (i.e., similar phase velocity) is seen in the wave group coming at ∼3.4 s. We interpret both of these wavegroups as predominat surface (Rayleigh) waves, possibly mixed with waves from the S-wave group. A very good waveform fit, besides a slight phase shift, can also be observed in Figure 10b, in the wave groups after 1.5 s associated with predominant Love waves. A similarly good matching, but for a different amplitude ratio (resulting in a lower apparent phase velocity), would be obtained for the direct S wave with the onset at ∼1.1 s. Similar conclusions, as mentioned in connection with Figure 10, can be deduced from Figure 11, except that the R13 waveform matching is considerably worse and that is why those waveforms were not considered for the phase velocity estimate in Table 4. The velocity estimate was derived for the peak values that are shown in Figure 11a between 2.1 s and 2.2 s, associated with the direct S wave (possibly mixed with waves from the P-wave group).

### 4.2. Directly Measured Versus Array-Derived Rotations

The triangular array that is described in the previous subsection offers the possibility to apply the ADR method in order to derive spatial gradients of displacement (or ground velocity) and, consequently, seismic rotation (or rotation rate). In its standard form, the method is based on finite differences of the records from the seismographs in the array, while assuming a plane wave passage and vanishing second-order gradients [29]. The method is described in detail, including the limitations of its applicability, in the companion paper [3]. In theory, the minimum number of stations for the ADR method is three, but practice has shown that it is worth deploying many more stations in order to compensate for small inconsistencies in deployment and site conditions, as well as inconsistencies in instrument responses—all of these cause errors that are assumed to effectively cancel out if a large number of stations is taken into account. Usually, certain formal quantitative conditions are set for the array aperture. Spudich and Fletcher [30] suggested that the horizontal extent of the array, parallel to the propagation direction, should not exceed one-quarter of the apparent wavelength. However, the basic and superior condition of ADR applicability is that the spatial gradients are uniform across the array, which follows from the plane wave assumption. Consequently, we should observe exactly the same waveforms (including their amplitudes), only time-shifted accordingly, from all of the instruments. When speaking about the spatial gradients, we should, perhaps, distinguish the real gradients from the measured ones. The uniformity of the measured gradients means that not only the plane wave assumption is reasonably satisfied (i.e., the radius of wavefront curvature is locally much larger than the prevailing apparent wavelength), but also the above-mentioned inconsistencies are negligible, in which case a smaller number of instruments would be satisfactory. The problem is that there is often no possibility to check the fulfillment of this condition prior to the ADR application (except for a relatively rare case of a rectangular array layout [3,5]), as even very tiny differences in the translational records (hardly visible in the seismograms) may lead to differences in spatial-gradient waveforms that are much more significant and, consequently, the ADR results are then not representative for the real gradients at the site. Fortunately, our triangular array does not consist of standard 3C seismographs, but of 6C Rotaphones also providing the rotation rate components, which allows for us to directly check whether the condition of uniform gradients (rotation rates) is satisfied.

Provided the ADR method is applicable, it enables us to verify our Rotaphone rotation rates by confronting them with the ADR results. It is frank to say that, in our case, the ADR method is, if applicable at all, at the very limit of its applicability for the following reasons: (1) the number of instruments exceeds the theoretical minimum of three only by one, (2) taking into account the fact that the apparent wavelength estimates in Table 4 are very rough, the wavelength may not be large enough with respect to the array aperture of 5 m, even for the dominant wave (especially for Expl5), let al.ne some other waves, and (3) for some of the directly measured rotation-rate components there can be no mention of their similarity between the different instruments (and, therefore, of uniform gradients), some are similar in shape of the waveforms, but differ in amplitudes to various extents. Although we are aware of these facts, we considered it to be useful to test the method and decide on the basis of its results to what extent it can potentially be used in order to verify our direct rotation-rate measurements.

It would be the most natural to compare the ADR results with the rotational records from R10, the instrument located at the center of the array. Unfortunately, it is this device that is disqualified from rotational measurements, due to the failure of one of the vertical geophones. In this situation, we have two options: either compare the ADR result with another Rotaphone from the array or to compare it with the average of measurements from the remaining three Rotaphones. Strictly speaking, none of the options is ideal. In the first one, records from the edge of the array may not be sufficiently representative for the entire array. In the second one, the averaging is performed over records shifted in time; we only hope that the time shifts, in our case, are small enough to affect the average insignificantly. Moreover, when comparing the ADR results with direct Rotaphone measurements, the provisional pre-calibration of individual geophones in the Rotaphone geophone systems is an issue, as it affects translational records, which are used in the ADR method, only very little if at all, but it can affect rotation rates from such deficiently calibrated instruments considerably more.

**Figure 12:**
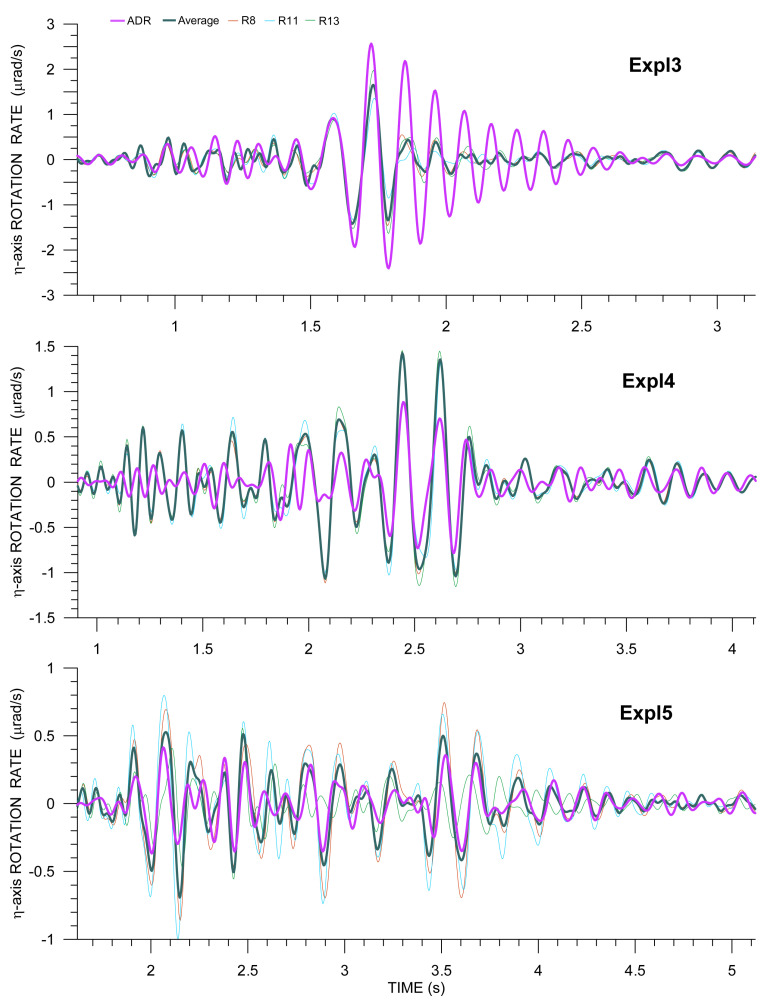
Comparison of array-derived rotation (ADR) transverse rotation rate (purple) with that from R8 (thin red), R11 (thin light blue), and R13 (thin green) and with the average transverse rotation (pine green) from those three instruments for explosions Expl3, Expl4, and Expl5. The transverse directions are perpendicular to the geometrical azimuths of 294${}^{\circ }$ (Expl3), 293${}^{\circ }$ (Expl4), and 285${}^{\circ }$ (Expl5).

**Figure 13:**
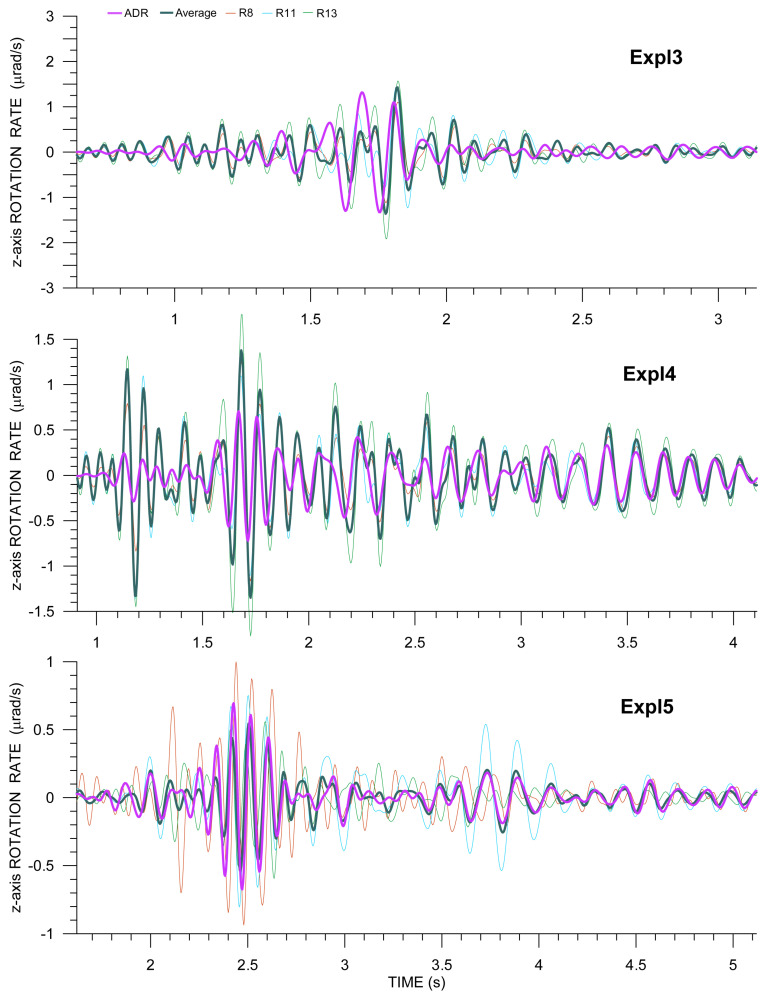
Comparison of ADR vertical rotation rate (purple) with that from R8 (thin red), R11 (thin light blue), and R13 (thin green), and with the average transverse rotation from those three instruments (pine green) for explosions Expl3, Expl4 and Expl5.

Examples of rotation rates from Rotaphones as compared with ADR results are shown in Figure 12 and Figure 13 for the transverse (η) and vertical (*z*) components, respectively, for all three explosions. The figures only display the S- and surface-wave windows in the seismograms, as the amplitudes of P-wave rotation rates are relatively small. When assessing the conformity of ADR results and directly measured rotation rates, it is not possible to consider whole seismograms, but only their parts corresponding to the different wave phases. The level of matching varies for different parts of the seismogram (and different explosions) mainly due to the fact that the conditions for the applicability of ADR (plane wave and uniform spatial gradient) are met by different waves to varying degrees, depending on the instantaneous period, current propagation direction, wave speed, etc. In the figures, better agreement can be observed for longer periods (longer wavelengths). Another general finding is that the waveform fit is no better for larger amplitudes (e.g., for Expl3); in our case, it is rather the opposite. In general, we obtain the best fit in the time intervals, where the relevant acceleration and rotation rate component waveforms match each other the best according to Equation (2) (that can be verified in Figure 10 and Figure 11). It means that, in those intervals, the plane wave assumption, the necessary condition of the ADR applicability, is acceptable.

In transverse components, we see the best fit for Expl3 between 1.5 and 1.6 s. For later peaks until 1.8 s, the agreement is also relatively good, perfect in phase and worse in amplitude. However, for most of those peaks, the difference between the averaged rotation rate and ADR rate is comparable with the spread of amplitudes from the individual Rotaphones. A very good matching is also seen for late Love wave arrivals, after 2.8 s. Analogous deductions can be made for Expl4 (for the dominant wave group between 2.3 and 2.8 s and the late Love wave arrivals after 3.5 s). In the case of Expl5, the spread of the directly measured values is quite large (i.e., spatial gradients are not uniform across the array). Yet, their average seems to be representative for the whole array, as it agrees relatively well with the ADR results for most of the seismogram depicted in Figure 12, bottom. Although the physical interpretation of this average is not obvious, its comparison with the ADR result may still be a good tool for verifying directly measured rotations.

A very bad mutual waveform agreement was obtained for the vertical component from Expl3. In Figure 12, top, a relatively good matching can only be observed for the Rayleigh Airy phase between 1.8 and 1.9 s, and then for some of the late Rayleigh wave phases after 2.7 s, which can be explained in a number of ways. For example, the wavefront may not be effectively planar for the closest explosion in this case. Another cause may be that the *z*-component is a superposition of two spatial gradients (Equation (1)) and so a relatively small error in both of them can have a greater impact on their sum and thus make the *z*-component less accurate. Or, perhaps, the average of directly measured rotation rates may not be as representative for the entire array as it is in some other cases, i.e., for Expl4 (relatively good waveform matching after 1.6 s and a very good fit after 3.1 s) and Expl5 (almost perfect waveform fit for the whole seismogram, despite the great variety of rotation rates from individual Rotaphones, both in terms of waveform shapes and their amplitudes).

## 5. Discussion and Conclusions

Four prototypes of the newest Rotaphone design, Rotaphone-CY, were involved in the GOF experiment. They underwent both the huddle and field-deployment testing. The results in the present study are regarded as preliminary due to only provisional and incomplete laboratory pre-calibration of the instruments. Nevertheless, the results are very promising and they indicate potentially good functionality of the instruments based on indirect and direct evidence.

**Figure 14:**
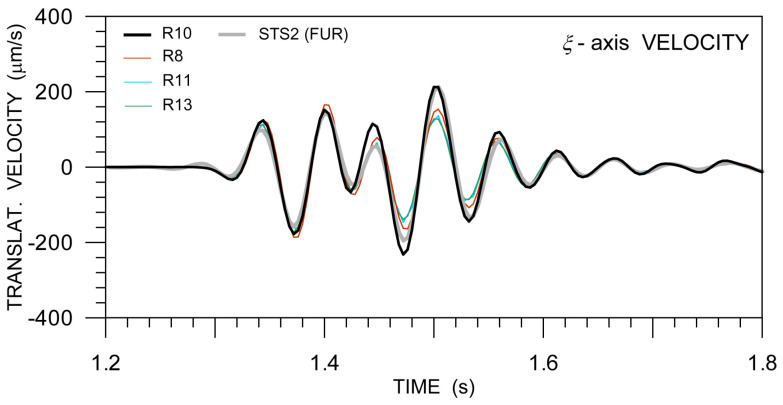
Radial ground velocity component $v_{\xi }$ from FUR, resampled to 250 Hz (gray) and from R10 (black). Records from R8 (red), R11 (light blue) and R13 (green), placed next to the concrete block, are shown for comparison. All of the records are filtered in the same way and instrument-corrected.

The huddle test took place in the shallow vault, in which the FUR broad-band station (German Regional Seismic Network) is situated. During the huddle test, all of the four prototypes (R8, R10, R11, and R13) were fully functional. One of them, R10, was installed on top of a concrete block that was detached from the vault building at a distance of about 60 cm from the STS2 sensor of the FUR station. The remaining three stood on the ground side by side along the longer side of the block, about 45 cm from each other at distances of 110–130 cm from R10. The translational records from all of the instruments slightly, but visibly differ, which we interpret as a result of deformation of the vault (normal modes) due to seismic wave passage. As expected, the records from R10 deviate the most, especially in the radial component (direction towards the explosion site). A comparison of the R10 records with the records from STS2 instrument at FUR, filtered to the same short-period range of 1–20 Hz (Figure 14), shows a good fit. Thus, although deviating so much from the others, we consider the R10 records to be reliable and we conclude that the differences reflect the different deployment conditions. Naturally, differences in translational components affect differences in rotational components (spatial gradients). Those mutual rotation-rate differences are even more significant; again, the most significant differences are observed for R10. Nothing implies that the R10 rotational records are incorrect. They look very similar to the records from the instruments installed on the same concrete block by other research groups, as shown by Bernauer et al. in this issue [28] (Figure 12 in that paper). Removing R10 from further considerations, mutual differences between rotational records from the other three Rotaphones can also be easily attributed to the normal modes of the vault. However, some of the Rotaphone rotational records match those from the other Rotaphones very well—examples may be Expl2 horizontal (ξ- and η axes) rotation rates from R8, R11, and R13 (the correlation coefficient reaches 0.974 in η-component) as well as Expl1 horizontal rotation rates from R8 and R11 (the correlation coefficient is 0.917 in ξ-component). We evaluate the Rotaphone-CY performance in the huddle test as very convincing.

In the field-deployment part of the GOF experiment, all of the Rotaphones were moved out of the vault and installed in a small-aperture triangular array with R10 at the center in order to record ground motions from three small-size explosions. Unfortunately, one of the vertical geophones of the R10 geophone system stopped working properly and it had to be excluded from the subsequent data processing. The exclusion of one geophone from R10 does not affect its translational records. However, it causes the rotational records, particularly rotation rates around horizontal axes (i.e., the ξ- and η-components), in order to display incorrect amplitudes.

The translational data that were produced by Rotaphones are undoubtedly correct, as they are based on direct measurement by geophones, standard short-period ground velocity sensors that have been used by seismologists in seismic prospecting and for various industrial applications for decades. The novelty of our approach lies in the measurements of rotational components that need to be checked (and that is why the GOF experiment was organized). Our 6C data offer the possibility to verify the rotational rates, at least in part, by matching them to the proper acceleration components measured at exactly the same point (not only measured by a seismograph standing nearby, as it is a common practice), as suggested by the R-TRs that are described by Equations (2) or (3). It is important to note that a poor or no fit does not imply that the rotational records are incorrect, because the real R-TRs in an inhomogeneous medium may differ significantly from those that are described by the given equations. On the other hand, a good fit means that (1) the real R-TRs at the site correspond well to those predicted by Equations (2) or (3) and (2) the relevant rotation waveforms are correct in shape. Thus, when a good fit is observed at least in parts of the seismograms, we can consider it to be an indication of correctly measured rotational components. One may, perhaps, argue that the rotational and acceleration components in question are not independent as it follows from the equations and, moreover, both originate from the same geophone measurement. However, the components are dependent only in theory: for a plane wave in a homogeneous structure. In reality, while taking the way they are deduced (averaging and simple time derivative vs. finite differencing within the geophone pairs) into account, they can be viewed as fully independent quantities and they can be used to verify the rotation rate data. A good match between the transverse rotation rate and vertical acceleration was found for the dominant wave from Expl2, dominant wave (mainly Rayleigh wave Airy phase), and later Rayleigh wave phases from Expl3 and Expl4, and most parts of seismograms of R8 and R11 from Expl5. Note that the appropriateness of the transverse rotational rate means that both horizontal components are correct, as the η-component comes from the original north and east components. Thus, the radial component is also reliable to the extent in which the transverse component matches vertical acceleration. The radial component could be either close to zero, as predicted by Equation (2) and seen in the case of Expl2, or it could be proportional to vertical velocity, as predicted by Equation (3). Indeed, although $v_{z}$ and $\Omega_{\xi }$ are not explicitly compared in the presented figures, it will not be difficult for the reader to see the shape similarity between the two components in the figures of 6C records, namely, in the wavetrains after 1.6 s from Expl3 (Figure 8) and after 2.3 s from Expl4 (Figure 9). All of these wavetrains belong to Rayleigh waves (including the Airy phases). The vertical rotation rate partly matches the transverse acceleration (as predicted by Equations (2) or (3), bottom) in the assumed Love-wave Airy phase and in later Love wave phases from Expl4 and Expl5 (Figure 10 and Figure 11), except R13 for Expl5.

In the cases of a good match of the waveforms that are predicted by Equation (2), it was possible to roughly estimate the apparent (along the Earth’s surface) phase velocity *c*. For this purpose, we used the $a_{z}/\Omega_{\eta }$ ratio. For the sake of the best accuracy, we restricted ourselves to the strongest waves in the $a_{z}$ seismograms and took the ratio for the acceleration peak value, because, at that time, the possible $v_{z}$-proportional term (Equation (3)) vanishes. This is one of the reasons why we preferred the $a_{z}/\Omega_{\eta }$ over the $a_{\eta }/\Omega_{z}$, as $v_{\xi }$ may not vanish at the peak times of $a_{z}$. In fact, the presence of $v_{\xi }$-proportional terms in the R-TRs is manifested by the noticeable phase shift between $\Omega_{z}$ and $a_{\eta }$ detected for all the explosions, see, e.g., Figure 5 (R10 only), Figure 10 and Figure 11. In order to obtain the most representative estimates of *c*, we calculated the mean value and the standard deviation from all of the Rotaphones in the huddle test and from R8, R11, and R13 in the field measurement. A slightly higher phase velocity estimate than expected for surface waves in unconsolidated sediments was obtained for Expl3. The reason may be that the dominant surface-wave phase is mixed with later arrivals of S waves at a near-normal incidence (e.g., reflected from the background). Such waves would naturally have a much higher apparent phase velocity. This hypothesis is consistent with the lower *c* estimate for the more distant Expl4. Another explanation may be that the transverse (η) rotation rate component, being defined with respect to the geometrical back azimuth of the explosion, is not actually the transverse one, i.e., the true back azimuth for the given seismogram phase is different. Based on our experience, the true back azimuth may change very rapidly in time. The hypothesis is supported by the relatively strong radial (ξ) rotation rate component of the wave in question, especially for R11. In principle, the apparent phase velocity can be also estimated from the time shifts between the individual Rotaphones in the array. However, the time shifts are very small, which significantly affects the accuracy of the estimate. The accuracy also decreases with decreasing amplitudes. Thus, we consider such an estimate as tolerably reliable, only for Expl3, for which it is 447 ± 133 m/s. The error intervals of this estimate and the estimate from the $a_{z}/\Omega_{\eta }$ ratio overlap substantially, which indirectly supports the conclusion that the horizontal rotation rates are measured correctly.

The criterion of the strongest wave was, in principle, met by different waves for different explosions. While it was the peak value in the surface wave group (predominantly the Airy phase) in the records from Expl3 and Expl4, for Expl5 we calculated the amplitude ratio for a wave in the S-wave group, probably the wave that is guided along the Earth’s surface. Taking into account the depth resolution of the rotation-rate based phase velocity estimates, which is approximately one wavelength [25,31,32], and the fact that we estimated the wavelength to be ∼30 m long for Expl5, then the estimated *c* is, in fact, approximately equivalent to the $v_{S30}$ parameter (average S-wave velocity for the topmost 30 m) used commonly in seismic engineering in order to classify soils. Our estimate of 311 ± 27 m/s corresponds to ground type C according to Eurocode 8 [33]. The stratigraphic profile of this ground typeis fully consistent with the geological setting at the site.

The main purpose of any comparative experiment is to compare the results from different instruments. In the present paper, we limit ourselves to only comparing records from the four Rotaphones-CY involved in the experiment, especially in its field-deployment phase. In the triangular array, despite the frequent overall similarities documented by relatively high correlation coefficient values, we observe small differences in amplitudes of translational components and more significant differences in amplitudes of rotational components, even when their waveforms are very similar (they are in phase). The differences in translational components vary from ∼2% in the records of Expl3 to ∼20% for Expl5. Such differences can be easily explained by local differences in structure beneath the individual Rotaphones. Even if we adopt the plane-wave approximation locally, such local inhomogeneities are copied into the effectively averaged structure parameters, thanks to the relatively short wavelength (in our case, the effect may be most pronounced for Expl5 because of the least wavelength estimate). Subsequently, maintaining the energy flux per unit area across the wavefront, a change in the effective wave velocity (or better, wave impedance, the product of velocity and density) by ∼1% leads to a change in amplitude of ground displacement (as well as velocity or acceleration) by ∼2% because the expression for the flux is quadratic in amplitude. We consider the anticipated local inhomogeneities that could be responsible for the observed differences in the translational amplitudes to be realistic. Rotational components (as spatial gradients) are much more sensitive to such local inhomogeneities than translational ones. The rotational components that were provided by Rotaphones in the array differed in peak amplitudes by 5% up to several tens of %, depending on the type of wave and explosion position. In fact, seismic rotational components can visibly change over a distance as short as, e.g., 2 m [3] or even shorter, as documented by other participants of the GOF experiment [34]. Firstly, this calls the frequent experimental arrangement of collocated rotational and translational measurements into question, where instruments stand side-by-side at some distance and not at exactly one point and, secondly, it reduces the informative value of comparative measurements targeted by the GOF experiment. On the other hand, differences in rotational components measured over a short distance may be a tool for detecting significant local inhomogeneities under the Earth’s surface.

The above-mentioned mutual differences in Rotaphone records indicate that the rotation rate (i.e., spatial gradient of ground velocity) is not uniform across the array. Its uniformity is an essential assumption on which the ADR method is based. Moreover, the central-position Rotaphone R10 was out of order for rotational measurements around the ξ- and η-axes. Thus, it is open to discussion as to whether to apply the method to our array measurement. We are inclined to think that this makes sense when comparing ADR with the average of rotation rates measured locally by R8, R11, and R13 Rotaphones. Both the ADR results and the average from local rotational measurements represent averaged rotation rates characteristic for the place occupied by the array but those quantities are independent given the way in which they were obtained. The Rotaphone measurements are based on differencing translational records from geophones that are placed very close to each other within one instrument (moving as a rigid body), which produces rotation rates that are then averaged over the array. In contrast, the ADR method is based on differencing translational records from geophones (with no rigid connection between them) that are mounted on instruments located much further apart (in our case by one order of magnitude) while implicitly assuming a plane wave passing through the array. The comparison of the directly measured rotations (or their average) with the ADR results is evaluated the same way as their comparison with the corresponding acceleration, see the discussion above. A good fit cannot be just a coincidence and it means that both the ADR and Rotaphone results should be correct. On the other hand, a bad or no fit does not necessarily imply the incorrectness of the Rotaphone rotational measurements, as there are many other factors at play. Despite the considerable handicaps of both methods (insufficient calibration on the one hand and application on the edge of applicability on the other), we obtained a surprisingly good match of results for some of the records or their parts. This is all the more surprising when considering that we work here in a high-frequency range (up to 15 Hz). As far as we know, comparisons of ADR with directly measured rotations published so far have been limited to frequencies that are well below 1 Hz [26,27], except for the companion paper [3]. Overall, we consider the comparison of rotational data from Rotaphones-CY with the ADR results to be very satisfactory.

To summarize, although the Rotaphones-CY that are involved in the GOF experiment have not yet been properly pre-calibrated in a laboratory equipped for the purpose (and so the results that are presented here have to be regarded as only preliminary), all four instruments passed the testing very well. The results suggest that their rotation rate records are roughly correct in the range up to 15 (or even 20) Hz. This assessment is based on both indirect indications (mutual similarity of their records or good correlation with the respective acceleration components) and, in part, on a direct comparison of Rotaphone rotational records with ADR. After the pre-calibration is completed, the measured rotations will only be much more accurate, no major changes in the records are expected.

The accuracy of the data from the properly pre-calibrated instruments is expected to be high enough to allow for much more accurate phase velocity estimates. Such estimates may reveal frequency surface-wave phase-velocity dispersion. We plan to revisit the measured 6C data from the GOF experiment and study these phenomena in detail, including, perhaps, inversion for shallow geological structure while using the method recently proposed by Málek et al. [35].

## Figures and Tables

**Table 1:** Rotaphone-CY specifications (only parameters relevant for the study).

	Translational Velocity	Rotation Rate
resolution	0.014 nm/s	0.042 nrad/s
hard-clip level	12.67 mm/s	31.68 mrad/s
frequency range	1–100 Hz ${}^{*}$	
sampling rate	250 Hz	
temperature range	−40–+70 °C	
cylinder diameter	44/55 cm (including handles)	
cylinder height	50 cm (without metal stems)	
weight	22 kg	

(*) Due to incomplete geophone pre-calibration at the time of writing this paper, the records are only reliable up to ∼20 Hz.

**Table 2:** Correlation coefficients of 6C records of overnight noise from Rotaphones R8, R10, R11, and R13. The coefficients for ground velocity along Z-, N-, and E- axes are shown above diagonals while those for rotation rates around each of the axes are shown below diagonals and highlighted in light gray.

	Z-axis				N-axis				E-axis			
Sensor	R8	R10	R11	R13	R8	R10	R11	R13	R8	R10	R11	R13
R8	1	0.968	0.985	0.983	1	0.989	0.990	0.987	1	0.980	0.981	0.977
R10	−0.109	1	0.975	0.961	0.432	1	0.988	0.989	0.363	1	0.976	0.975
R11	0.161	0.212	1	0.97	0.344	0.390	1	0.989	0.196	0.452	1	0.978
R13	0.117	−0.111	−0.283	1	0.119	0.124	−0.043	1	0.213	−0.015	−0.101	1

**Table 3:** Huddle-test active-source Rotaphone measurements by component (*z*—vertical, ξ—radial, η—transverse): means over all Rotaphones ± standard deviation. Key: $v_{i}$ and $\Omega_{i}$, i=z,ξ,η, are maximum absolute values of translational and rotational components, respectively; SNR${}_{T}$ and SNR${}_{R}$ are signal-to-noise ratios in translational and rotational components, calculated from noise in time interval between −10 s and −2 s prior the first onset; $C_{T}$ or $C_{R}$ represent Pearson correlation coefficients between translational or rotational records from each two Rotaphones; $f_{p}$ means the prevailing frequency, calculated as instantaneous frequency of maximum rotation rate; *c* denotes apparent phase velocity calculated as the $a_{z}/\Omega_{\eta }$ ratio at the $\Omega_{\eta }$ maximum; $\lambda_{p}$ is the estimate of the prevailing apparent wavelength (along the Earth’s surface) calculated whiel using $a_{z}/\Omega_{\eta }$ ratios and $f_{p}$, both for maximum $\Omega_{\eta }$.

		z	ξ	η
**Expl1**	$v_{i}$ [μm/s]	22.9 ± 0.6	16.3 ± 1.1	15.2 ± 0.6
	SNR${}_{T}$	44 ± 3	36 ± 3	34 ± 3
	$C_{T}$	0.948 ± 0.041	0.951 ± 0.017	0.961 ± 0.021
	$\Omega_{i}$ [μrad/s]	2.4 ± 1.2	2.6 ± 1.0	3.0 ± 2.3
	SNR${}_{R}$	54 ± 32	39 ± 19	59 ± 25
	$C_{R}$	0.143 ± 0.355	0.612 ± 0.206	0.782 ± 0.081
	$f_{p}$ [Hz]	15.8 ± 1.4	11.7 ± 2.4	12.6 ± 1.1
	*c* [m/s]	N.A.		
	$\lambda_{p}$ [m]	N.A.		
**Expl2**	$v_{i}$ [μm/s]	418.6 ± 18.6	191.9 ± 27.3	55.2 ± 7.3
	SNR${}_{T}$	1617 ± 87	753 ± 104	224 ± 34
	$C_{T}$	0.998 ± 0.001	0.977 ± 0.014	0.981 ± 0.011
	$\Omega_{i}$ [μrad/s]	21.5 ± 11.2	13.4 ± 3.6	145.7 ± 33.6
	SNR${}_{R}$	856 ± 661	257 ± 77	1064 ± 703
	$C_{R}$	−0.099 ± 0.377	0.156 ± 0.383	0.689 ± 25
	$f_{p}$ [Hz]	16.0 ± 0.1	15.3 ± 1.4	16.6 ± 0.205
	*c* [m/s]	264 ± 61		
	$\lambda_{p}$ [m]	16 ± 4		

**Table 4:** Field active-source Rotaphone measurements by component (*z*—vertical, ξ—radial, η—transverse): means over Rotaphones ± standard deviation. Key: see caption of Table 3; $C_{T}^{\prime }$ or $C_{R}^{\prime }$ are maxims of correlation function between Rotaphone records.

		z	ξ	η
**Expl3**	$v_{i}$ [μm/s]	17.4 ± 0.4	14.0 ± 0.3	17.4 ± 0.5
	SNR${}_{T}$	53 ± 3	53 ± 1	51 ± 4
	$C_{T}$	0.998 ± 0.001	0.997 ± 0.002	0.998 ± 0.001
	$C_{T}^{\prime }$	0.998 ± 0.001	0.998 ± 0.002	0.998 ± 0.001
	$\Omega_{i}$ [μrad/s]	1.5 ± 0.3	1.4 ± 0.3 ${}^{*}$	1.7 ± 0.2 ${}^{*}$
	SNR${}_{R}$	65 ± 40	69 ± 19 ${}^{*}$	52 ± 22 ${}^{*}$
	$C_{R}$	0.752 ± 0.147	0.947 ± 0.019 ${}^{*}$	0.926 ± 0.028 ${}^{*}$
	$C_{R}^{\prime }$	0.775 ± 0.132	0.957 ± 0.012 ${}^{*}$	0.940 ± 0.029 ${}^{*}$
	$f_{p}$ [Hz]	10.3 ± 0.7	8.8 ± 0.5	7.3 ± 0.4
	*c* [m/s]	518 ± 85 ${}^{*}$		
	$\lambda_{p}$ [m]	51 ± 8 ${}^{*}$		
**Expl4**	$v_{i}$ [μm/s]	10.4 ± 0.2	9.6 ± 0.1	11.6 ± 0.2
	SNR${}_{T}$	29 ± 2	25 ± 1	31 ± 2
	$C_{T}$	0.998 ± 0.002	0.997 ± 0.002	0.997 ± 0.002
	$C_{T}^{\prime }$	0.998 ± 0.002	0.997 ± 0.002	0.997 ± 0.002
	$\Omega_{i}$ [μrad/s]	1.6 ± 0.3	1.0 ± 0.2 ${}^{*}$	1.4 ± 0.1 ${}^{*}$
	SNR${}_{R}$	44 ± 18	32 ± 2 ${}^{*}$	39 ± 9 ${}^{*}$
	$C_{R}$	0.891 ± 0.046	0.951 ± 0.006 ${}^{*}$	0.958 ± 0.021 ${}^{*}$
	$C_{R}^{\prime }$	0.892 ± 0.045	0.959 ± 0.001 ${}^{*}$	0.967 ± 0.016 ${}^{*}$
	$f_{p}$ [Hz]	10.7 ± 0.1	9.6 ± 1.1	6.3 ± 0.4
	*c* [m/s]	406 ± 110 ${}^{*}$		
	$\lambda_{p}$ [m]	38 ± 10 ${}^{*}$		
**Expl5**	$v_{i}$ [μm/s]	5.2 ± 0.6	6.4 ± 0.2	11.4 ± 0.5
	SNR${}_{T}$	20 ± 3	26 ± 2	53 ± 4
	$C_{T}$	0.966 ± 0.017	0.964 ± 0.018	0.978 ± 0.014
	$C_{T}^{\prime }$	0.986 ± 0.004	0.976 ± 0.013	0.993 ± 0.004
	$\Omega_{i}$ [μrad/s]	0.8 ± 0.2	1.1 ± 0.1 ${}^{*}$	0.8 ± 0.2 ${}^{*}$
	SNR${}_{R}$	36 ± 18	49 ± 7 ${}^{*}$	25 ± 2 ${}^{*}$
	$C_{R}$	0.204 ± 0.489	0.805 ± 0.058 ${}^{*}$	0.519 ± 0.262 ${}^{*}$
	$C_{R}^{\prime }$	0.603 ± 0.167	0.813 ± 0.062 ${}^{*}$	0.550 ± 0.265 ${}^{*}$
	$f_{p}$ [Hz]	9.4 ± 1.2	10.1 ± 1.4	6.8 ± 1.2
	*c* [m/s]	311 ± 27 ${}^{†}$		
	$\lambda_{p}$ [m]	33 ± 3 ${}^{†}$		

(*) Only R8, R11 and R13 are taken into account. (^†^) Only R8 and R11 are taken into account.

## Data Availability

The study is based on preliminary data (as several times explicitely mentioned) which are not publicly available.

## Abbreviations

The following abbreviations are used in this manuscript:

6C	Six-component
ADR	Array-derived rotation
ASL	Albuquerque Seismological Laboratory
GOF	Geophysical Observatory in Fürstenfeldbruck
R-TRs	Rotation-to-translation relations
SNR	Signal-to-noise ratio

## Appendix A. The GOF Experiment Explosion Summary

Although all relevant information on the explosions considered in the present study is provided in the main text, the authors anticipate papers on the GOF experiment by other authors, and so a summarizing table of all explosions is provided here (Table A1) for purposes of easier identification of the explosions allowing possible comparison of our results with the results of others.

**Table A1:** A list of explosions used as active sources in the GOF experiment. Explosion time is specified both in Central European Time (CET) and Coordinated Universal Time (UTC). Azimuth and Distance refer to Rotaphone R10. Note that R10 was moved between Expl2 and Expl3 from the vault (48.162899 N, 11.2752 E) to the field deployment site (48.1628313 N, 11.27521977 E).

Explosion	Day	CET	UTC	Latitude	Longitude	Explosive	Azimuth	Distance
		h:m	h:m:s	N, deg	E, deg	Amount [g]	from N [${}^{\circ }$]	[m]
Expl1	19 November 2019	11:26	N.A.	48.16480388	11.27600664	150	15.77	220
Expl2	19 November 2019	16:16	15:16:45.09	48.16317391	11.27462563	500	305.66	52
Expl3	20 November 2019	14:18	13:18:37.86	48.16448354	11.26965715	1500	294.01	452
Expl4	20 November 2019	14:46	13:46:16.39	48.16520805	11.26683228	1500	293.02	676
Expl5	20 November 2019	15:17	14:17:44.38	48.16517405	11.26192905	1500	284.81	1020
